# Community detection in empirical kinase networks identifies new potential members of signalling pathways

**DOI:** 10.1371/journal.pcbi.1010459

**Published:** 2023-06-23

**Authors:** Celia De Los Angeles Colomina Basanta, Marya Bazzi, Maruan Hijazi, Conrad Bessant, Pedro R. Cutillas

**Affiliations:** 1 Cell signaling and Proteomics Group, Centre for Genomics and Computational Biology, Barts Cancer Institute, Queen Mary University of London, London, United Kingdom; 2 Warwick Mathematics Institute, University of Warwick, Coventry, United Kingdom; 3 The Alan Turing Institute, London, United Kingdom; 4 School of Biological and Chemical Sciences, Queen Mary University of London, London, United Kingdom; University of Virginia, UNITED STATES

## Abstract

Phosphoproteomics allows one to measure the activity of kinases that drive the fluxes of signal transduction pathways involved in biological processes such as immune function, senescence and cell growth. However, deriving knowledge of signalling network circuitry from these data is challenging due to a scarcity of phosphorylation sites that define kinase-kinase relationships. To address this issue, we previously identified around 6,000 phosphorylation sites as markers of kinase-kinase relationships (that may be conceptualised as network edges), from which empirical cell-model-specific weighted kinase networks may be reconstructed. Here, we assess whether the application of community detection algorithms to such networks can identify new components linked to canonical signalling pathways. Phosphoproteomics data from acute myeloid leukaemia (AML) cells treated separately with PI3K, AKT, MEK and ERK inhibitors were used to reconstruct individual kinase networks. We used modularity maximisation to detect communities in each network, and selected the community containing the main target of the inhibitor used to treat cells. These analyses returned communities that contained known canonical signalling components. Interestingly, in addition to canonical PI3K/AKT/mTOR members, the community assignments returned TTK (also known as MPS1) as a likely component of PI3K/AKT/mTOR signalling. We drew similar insights from an external phosphoproteomics dataset from breast cancer cells treated with rapamycin and oestrogen. We confirmed this observation with wet-lab laboratory experiments showing that TTK phosphorylation was decreased in AML cells treated with AKT and MTOR inhibitors. This study illustrates the application of community detection algorithms to the analysis of empirical kinase networks to uncover new members linked to canonical signalling pathways.

## Author summary

Kinases are key enzymes that regulate the transduction of extracellular signals from cell surface receptors to changes in gene expression via a set of kinase-kinase interactions and signalling cascades. Inhibiting hyperactive kinases is a viable therapeutic strategy to treat different cancer types. Unfortunately for cancer therapy, kinase signalling networks are robust to external perturbations, thus allowing tumour cells to orchestrate mechanisms that compensate for inhibition of specific kinases. Therefore, there is a need to better understand kinase network structure to identify new therapeutic targets. Here, we reconstructed kinase networks from phosphoproteomics data, and compared the activity of its kinase interactions in acute myeloid leukaemia (AML) cells. We then tested community detection algorithms to identify kinase components associated with PI3K/AKT/mTOR signalling, a paradigmatic oncogenic signalling cascade. We found that TTK was usually grouped with networks derived for PI3K, AKT and mTOR kinases. Wet-lab experiments confirmed that TTK is likely to act downstream of AKT and mTOR. We thus showed that our methods can be used to identify potential new members of canonical kinase signalling cascades.

## Introduction

Cells respond to changes in their environment through kinase-driven biochemical pathways that regulate the flux of signalling from cell surface receptors to changes in the expression levels of genes involved in cell functions [1]. Signal transduction is therefore essential to the upkeep of many biological processes including immune function [2], ageing/senescence [3], and growth, amongst others. Conversely, the dysregulation of signalling pathways can result in a range of different pathologies, such as metabolic syndrome, autoimmune diseases, or cancer [4,5]. Sustained proliferative signalling brought about by genetic mutations or by aberrant expression or activation of tumour suppressor genes and proto-oncogenes is a key cancer hallmark. For this reason, kinases are one of the most widely pursued targets for cancer therapeutics.

Although approaching signalling as a set of pathways is useful to conceptualise some of its properties, it is now recognized that signalling pathways form complex networks of interactions and enzymatic reactions [6]. A network in its simplest form is a graph, where nodes represent entities of interest and edges represent pairwise interactions between such nodes [7]. Quantitative phosphoproteomics can provide new insights into these complex kinase signalling networks through the measurement of kinase phosphorylation, and capture the complexity of interactions within and between kinase signalling pathways at the network level [8,9]. While quantitative phosphoproteomics have been widely used to study changes in kinase activities in cancer cells [10–14], studies that incorporate quantitative phosphoproteomics with kinase-substrate networks are fewer and relatively recent [8,9,15–17]. A problem has been the lack of phosphorylation sites that are markers of kinase network circuitry and which therefore allow for cell-type specific reconstruction of signalling networks from phosphoproteomics data. To address this issue, Hijazi *et al* recently described a set of 6,000 phosphorylation sites that define kinase-kinase interactions [16], from which networks may be reconstructed in a given cellular state.

Here, we aimed to characterise novel kinase interactions in canonical cancer signalling pathways. To do so, we construct networks of kinase-kinase relationships with edge weights defined by the change in the levels of markers of kinase-kinase relationships in response to the inhibition of a given kinase (as measured by quantitative phosphoproteomics). Once networks are constructed for each perturbation, we applied the community detection approach modularity maximisation, which has been used in a wide variety of biological networks [18] and has achieved favourable performance in comparative studies on biological networks [19]. In doing so, our aim is to illustrate how one can use community detection techniques from network science to analyse signalling derived from quantitative phosphoproteomics, and to demonstrate that studying the mesoscale structure (e.g., community structure) [7,20] of the resulting network can reveal novel biological insights about cancer kinase signalling pathways.

## Materials and methods

### Data description

We first analysed enrichment values of 1500 kinase-kinase interactions in P31/FUJ cells treated separately with the kinase inhibitors trametinib, GDC0994, GDC0941 and AZD5363, measured by z-score analysis as in the Kinase Substrate Enrichment Analysis (KSEA) method [10] (these values were taken from four of the columns in the Supplementary dataset 5 published in [16]). The kinase-kinase interactions were defined in previous work [16] based on the existence of common putative downstream targets (PDTs), i.e. both kinases in a kinase-kinase relationship that define a given edge act upstream of a number of phosphorylation sites. We focus on kinase-kinase relationships inhibited by the treatments (i.e., with negative z-score values) where one has some notion of ground truth for the pathway containing the main target of the inhibitor. That is, MAP2K1 and MAPK1/3, the respective targets of trametinib and GDC0994, belong to the MEK/ERK pathway [21], while PIK3CA and AKT1/2, the respective targets of GDC0941 and AZD5363, belong to the PI3K/AKT/mTOR pathway [3]. Table 1 shows summary statistics for the dataset portion that we tackle in the present paper, which focuses on the analysis of the GDC0941 and AZD5363 treatments, while that of trametinib and GDC0994 treated cell measurements can be found in the Supplementary Information.

### Network construction

In this study, we work under the hypothesis that the inhibition of a kinase that belongs to a pathway will result in the inhibition of its downstream kinase interactions within that pathway. For each treatment, we constructed a network in which the edges are the pairwise kinase interactions with negative z-scores (i.e., inhibited) and the nodes are the kinases that are part of said interactions using custom-made Python scripts (made publicly available at https://github.com/celiaccb/KinasesCommunityDetection as part of this work), resulting in four weighted and undirected networks (i.e., without assumption on whether the kinases act upstream or downstream of each other). Additionally, we constructed networks from the pair kinase interactions with positive z-scores (i.e., upregulated), as detailed on the S3 Appendix. We adopt this approach because the presence of a given edge (kinase-kinase interaction) in the network would be revealed by the decrease in phosphorylation sites that define such edges as a result of treatment with specific kinase inhibitors.

Loosely speaking, a community in a network is a set of nodes that are “more densely” connected to each other than they are to nodes in the rest of the network [20]. Since we are interested in capturing the kinase interactions with the highest decrease in activity (i.e., lowest z-scores) in response to a treatment, we use the absolute value of z-scores as the weights of the kinase-kinase edges. The weights of the kinase-kinase edges is then proportional to the extent of inhibition that the kinase-kinase interaction represents experiences with said treatment (compared to control).

**Table 1:** Summary statistics for strictly negative z-scores.

Treatment	No. of kinases	min	max	*μ*	*σ*
trametinib	69	-3.732	-0.000607	-0.278	0.276
GDC0941	72	-6.749	-0.00178	-0.590	0.779
AZD5363	74	-16.886	-0.00334	-0.505	0.966
GDC0994	67	-1.411	-0.00233	-0.309	0.261

Data available in [16] (see Data description). The column “min” (resp., “max”) corresponds to the smallest (resp., largest) strictly negative z-score for the corresponding treatment. The columns *μ* and *σ* correspond to the mean and standard deviation, respectively, of strictly negative z-score values for each treatment.

### Community detection

We denote by *n* the number of nodes in a network and by ***A*** the corresponding adjacency matrix. In this paper, the nodes are kinases and the entries of ***A*** are the absolute value of negative z-scores (see Network construction). We use *modularity maximisation* to identify communities. The modularity maximisation problem can be stated as follows:

$$\underset{C\in C}{\max}\overset{}{\underset{}{\;\sum_{i,j=1}^{n}\left(A_{ij}-P_{ij}\right)\delta \left(c_{i},c_{j}\right)}}$$ (1)

where *C* is the set of all *n*-node partitions, ***A*** is the adjacency matrix of the observed network, ***P*** is an expected null network under some null model, and *c*_*i*_ is the set assignment of node *i* [20,22]. This method partitions a network into sets of nodes called “communities” that have a larger total internal edge weight than that expected in the same sets in a null network, generated from some null model.

**Fig 1:**
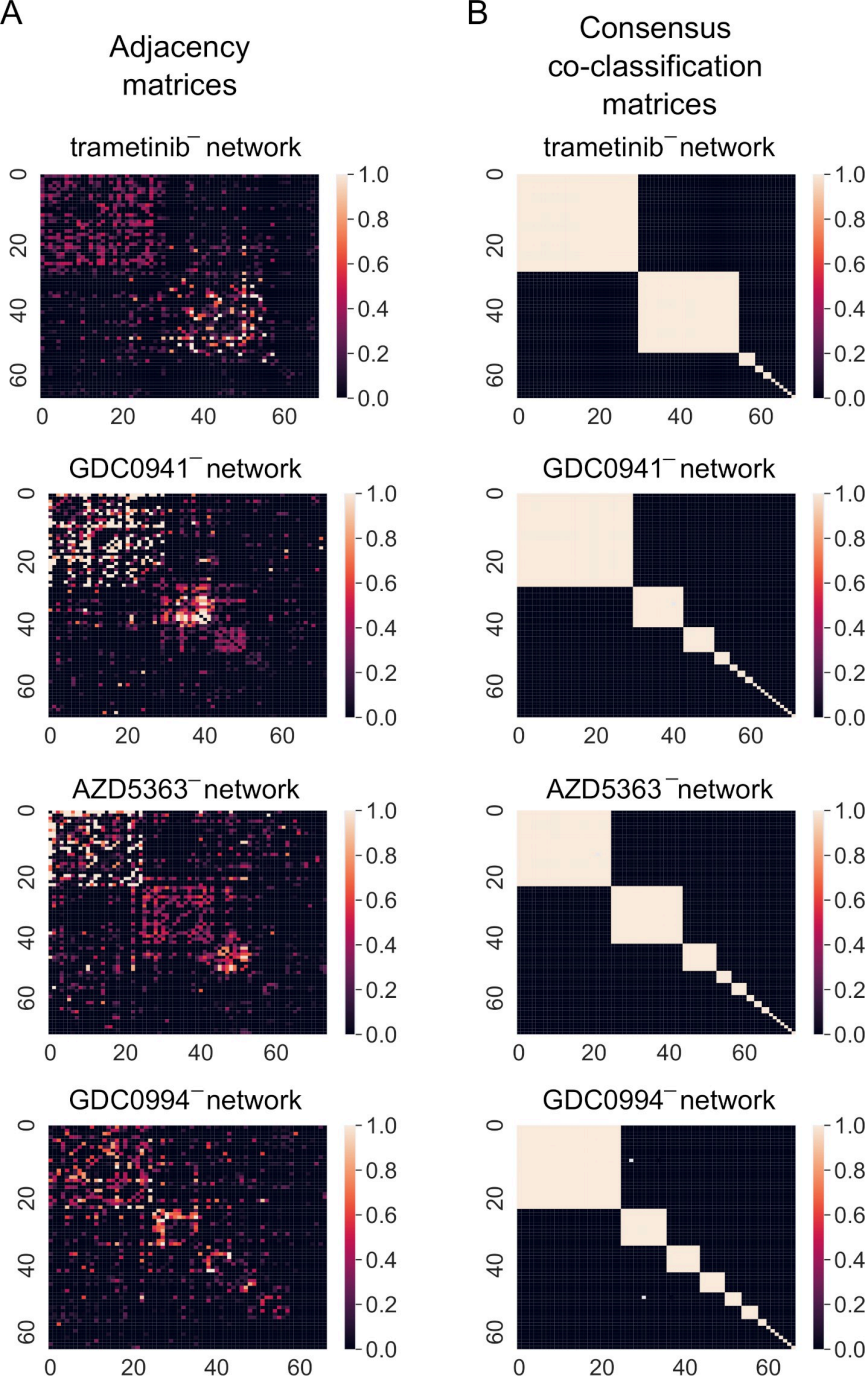
Results of community detection from phosphoproteomics-derived networks. Adjacency matrix (panel A) and consensus co-classification matrix (panel B). In panel A, we show the adjacency matrix of each kinase network ordered by their community assignment in the consensus partition. We cap the entries of the adjacency matrix at 1 to emphasize structure. In panel B, we show the co-classification matrix of a consensus partition (see Community detection), which takes the values 1 or 0. The colors scale with the entries of the matrices.

To find locally optimal partitions for the community detection approach modularity maximisation in Community detection, we use the locally greedy algorithmic heuristic Louvain [23]. We consider both a“uniform” and “Newman-Girvan” null network [24], and choose the uniform null network for our experiments on the basis of slightly better alignment with “ground truth” canonical pathways (see Results) across different algorithmic runs. We use a nondeterministic version of Louvain in which node order is randomised at the start of each iteration. This stochasticity can yield different partitions across different runs. To compute a “consensus partition”, we use the iterative approach in [25]. We obtain an ensemble of partitions using the Louvain algorithm (the size of the ensemble is the number of algorithmic runs), for which we compute a “co-classification matrix”. We reiterate our community detection procedure on the co-classification matrix until the new co-classification matrix resulting from the corresponding partition ensemble is binary (typically after 1 or 2 iterations). The partitions we obtain are robust (e.g., to different repetitions of the consensus procedure), see S1 Appendix. We show adjacency matrices and corresponding co-classification matrices in Fig 1.

### Wet-lab experiments: Phosphoproteomics analysis of kinase inhibitor-treated cells

To experimentally verify novel computational findings, we carried out new phosphoproteomics analyses following the protocol described in the S4 Appendix and previous work [16,26]. We chose the P31/Fuj cell line to validate observations related to the PI3K/AKT/mTOR pathway because this cell line shows an increase in the activity of the PI3K/AKT/mTOR pathway due to an inactivating mutation in the PTEN. A lipid phosphatase that negatively regulates PI3K activity. Briefly, the P31/FUJ cell line was grown in RPMI medium supplemented with 10% FBS and 1% Pen/Strep. Cells were treated with 1 *μ*M CFI-402257 (TTKi), MK2206 (AKTi), BYL719 (PI3Ki) or AZD8055 (MTORi) for three hours, in order to give enough time for the TTKi CFI-402257 to reach TTK in the cell nucleus. Table 2 shows the supplier and catalogue number of the compounds.

**Table 2:** Additional information on the compounds used.

Name	Intended Target	Supplier	Catalog No.
CFI-402257	TTK	MedChemExpress	HY-101340
MK2206	AKT	Selleckchem	S1078
BYL719	PI3K	Selleckchem	S2814
AZD8055	mTOR	Selleckchem	S1555

After treatment, cells were lysed in urea buffer and digested with trypsin. Finally, phosphopeptides were enriched using TiO2 and analysed in a LC-MS/MS system, which consisted of an Ultimate 3000 ultra-high-pressure chromatography connected to a Q-Exactive Plus mass spectrometer. Peptide identification and quantification was performed using Mascot search engine and Pescal, respectively, as described in [26]. The mass spectrometry proteomics data has been deposited to the ProteomeXchange Consortium via the PRIDE partner repository [27] with the dataset identifier PXD026039.

## Results

### Community structure can reflect kinase signalling pathways

The main aim of this study is to assess whether the application of community detection techniques to quantitative phosphoproteomics based kinase networks can identify new members of canonical kinase signalling pathways. For this purpose, we first assess whether known kinases in the signalling pathway PI3K/AKT/mTOR belong to the same community. That is, we compare the results of community detection with what we regard as “ground truth” in the present context.

Two networks were constructed based on the edge enrichment of kinase-kinase relationships (see Network construction), measured as z-scores, of AML cells treated separately with the kinase inhibitors GDC0941 (a PIK3CA/PI3K inhibitor) and AZD5363 (AKT inhibitor). We refer to these networks as GDC0941 and AZD5363, respectively, for the remainder of the paper, where the superscript “^–^” is to emphasise that we only consider negative z-scores (i.e., kinase-kinase relationships that were inhibited by the compounds). Furthermore, in each network we only focus on the community containing the main target of the inhibitor the cells were treated with. For example, the communities containing PI3K kinase alpha isoform (gene name PIK3CA) and AKT1/2 (AKT isoforms 1 and 2 are considered as one since they have the same z-scores due to having the same putative downstream targets assignments) were selected for networks GDC0941^–^ and AZD5363^–^ , respectively, and we denote these respective communities by GDC0941^–^_(PIK3CA)_ and AZD5363^–^_(AKT1/2)_ .

**Fig 2:**
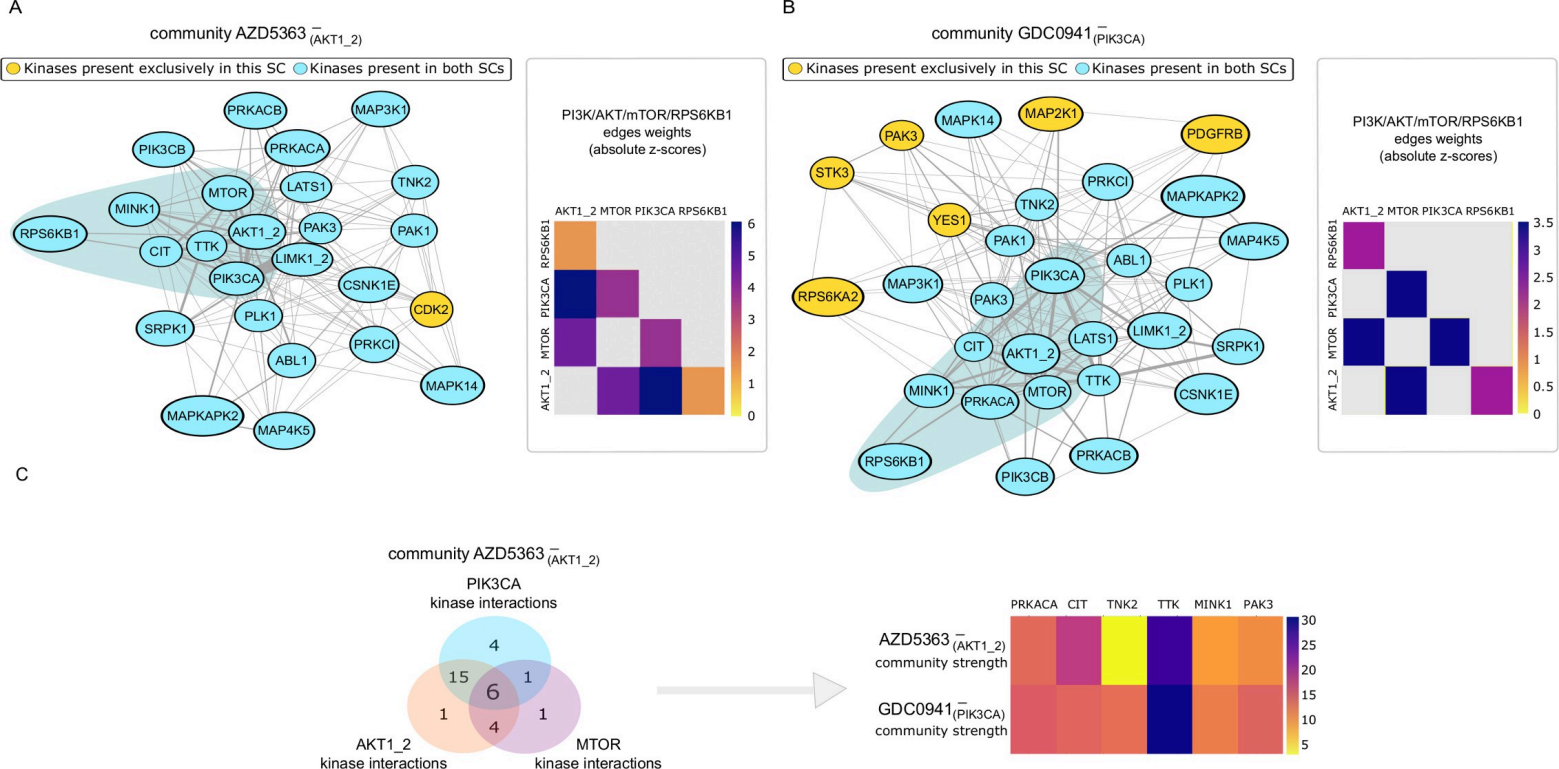
Identification of canonical and non-canonical kinases in PI3K/AKT networks. A. Kinase interactions of the selected communities in network AZD5363^–^ . Abbreviations: SC; selected community. On the left hand side of panel A the nodes and edges within the community AZD5363^–^_(AKT1/2)_ are depicted, in which the yellow nodes represent kinases present in AZD5363^–^_(AKT1/2)_ but not GDC0941^–^_(PIK3CA)_ , and the blue ones represent the nodes present in both communities. B. Kinase interactions of the selected communities in network GDC0941^–^_(PIK3CA)_ . On the left hand side of panel B the nodes and edges within the community GDC0941^–^_(PIK3CA)_are depicted, in which the yellow nodes represent kinases present in GDC0941^–^_(PIK3CA)_ but not in AZD5363^–^_(AKT1/2)_, and the blue ones represent the nodes that are present in both communities. The heatmaps on the right hand of panel A and B show the weights of the edges between well-known members of the PI3K/AKT/mTOR pathway in each of the communities, in which a grey cell indicates that interaction is not present in that community. C. Kinase interactions and community strength show TTK is likely to be downstream of PI3K/AKT/mTOR signalling. The venn diagram shows the intersections between the PIK3CA, AKT1/2 and MTOR edges/kinase interactions in the AZD5363^–^_(AKT1/2)_ community, in which six kinases have direct interactions with PIK3CA, AKT1/2 and MTOR. In panel B, the community strength (i.e. the sum of the weight of the edges between a node and other nodes in its community) of each of the said six kinases in the AZD5363^–^_(AKT1/2)_ and GDC0941^–^_(PIK3CA)_ communities is depicted.

PIK3CA and AKT1/2 are two of the main members of the PI3K/AKT/mTOR signalling pathway, which is often activated in a range of cancers and contributes to their development [28]. Thus, we expect the selected communities in networks GDC0941^–^ and AZD5363^–^ to be similar or identical in terms of content, and a reflection of the kinase interactions in this pathway. We found that the communities GDC0941^–^_(PIK3CA)_ and AZD5363^–^_(AKT1/2)_ share 24 kinases (blue nodes in Fig 2A and 2B) while CDK2 is part of the community AZD5363^–^_(AKT1/2)_ (yellow node in Fig 2A) but not GDC0941^–^_(PIK3CA)_. Six other kinases were part of GDC0941^–^_(PIK3CA)_ but not AZD5363^–^_(AKT1/2)_ (purple nodes in Fig 2B), four of which we attribute to MAP2K1 signalling (further discussed in S2 Appendix). Of note, PIK3CA, AKT1/2, mTOR and RPS6KB1, which are well known members of the canonical PI3K/AKT/mTOR signalling pathway [28], are present in both communities, which is concomitant with the fact that the edges between these kinases have large weight values (see heatmaps in Fig 2A and 2B) in both networks (i.e. their activity is highly decreased in response to both inhibitors).

Thus, the community detection algorithm used in this study returned the PI3K/AKT/mTOR canonical signalling pathway, suggesting that this approach can identify biologically meaningful associations. We stress, however, that community detection alone is hypothesis generating and not sufficient for the unambiguous identification of novel members in said pathways, for which we delineate further steps in the next two sections.

### TTK is a likely downstream target of PI3K/AKT/mTOR signalling

We next investigated which of the kinases placed in the communities AZD5363^–^_(AKT1/2)_ and GDC0941^–^_(PIK3CA)_ are most likely to act downstream of PI3K/AKT/mTOR signalling. To do so, we narrowed the list of 24 kinases assigned to both the GDC0941^–^_(PIK3CA)_ and AZD5363^–^_(AKT1/2)_ communities (see Community structure can reflect kinase signalling pathways) based on two criteria. First, we discarded all kinases that do not have a direct edge with all three kinases PIK3CA, AKT1/2 and mTOR in the AZD5363^–^ network. If a kinase does not have a direct relationship with all three kinases in PI3K/AKT/mTOR signalling (see Network construction) it is more likely to act downstream of some but not all kinases, since it either means that the kinase does not share downstream targets with PI3K, AKT and mTOR, or that the relationship is not included in the network because its activity is unchanged or increased in response to the different inhibitors.

We found that, in addition to the known PI3K/AKT/mTOR pathway members AKT1/2, mTOR and RPS6KB1, six kinases have direct edges with PIK3CA, AKT1/2 and mTOR in the AZD5363^–^_(AKT1/2)_ community, as shown in Fig 2C. Secondly, we computed the *community strength* (CS) (i.e., the sum of the weight of the edges between a node and other nodes in its community) of said kinases in GDC0941^–^_(PIK3CA)_ and AZD5363^–^_(AKT1/2)_ (see Fig 2C). Interestingly, TTK had the highest absolute CS by far out of the six selected kinases in both communities, as well as the third highest absolute CS of all kinases in GDC0941^–^_(PIK3CA)_ and the fourth highest absolute CS of the AZD5363^–^_(AKT1/2)_ kinases, meaning its interactions with the other kinases in both communities have the highest decrease in activity in response to both PIK3CA and AKT1/2 inhibition. We observed similar results on breast cancer cells treated with rapamycin and oestrogen, as detailed on the S2 Appendix under the supporting information. These results strongly suggest that TTK is likely associated with PI3K/AKT/mTOR signalling across different types of cancers.

The Citron Rho-interacting (CIT) kinase has the second highest absolute CS in AZD5363^–^_(AKT1/2)_ but the fourth highest absolute CS in GDC0941^–^_(PIK3CA)_. The AZD5363 inhibitor has previously been shown to significantly decrease the activity of kinases ROCK1 and ROCK2 [29], which share with CIT the upstream effectors RHOA and RHOC [30]. Therefore, we propose that AZD5363 might decrease CIT activity to a higher degree than GDC0941 through the inhibition of RHOA and RHOC, although the validity of this hypothesis needs to be further investigated.

PRKACA has the third highest absolute CS in AZD5363^–^_(AKT1/2)_ and the third highest absolute CS in GDC0941^–^_(PIK3CA)_. Moody et al previously found that PRKACA drives resistance to HER2 inhibitors in breast cancer by promoting cell survival through the inactivation of the pro-apoptotic protein BAD, a mode of action shared with AKT. Additionally, they observed that increased expression of PRKACA did not salvage phosphorylation of AKT or mTOR [31]. Based on these observations, PRKACA may also be a downstream target of PI3K/AKT/mTOR signalling in some contexts.

### Phosphoproteomics analysis provides supporting evidence for TTK being phosphorylated downstream of PI3K/AKT/mTOR signalling

As shown in the previous section, the kinase TTK–a mitotic kinase also known as MPS1 –was found to be in the same community as PI3K, AKT and mTOR, suggesting that TTK may be upstream or downstream of the PI3K/AKT/mTOR pathway. To validate this finding, the cell line P31/FUJ was treated with 1 *μ*M CFI-402257 (a highly specific small molecule inhibitor of TTK [32], thereafter named TTKi), MK2206 (AKTi), BYL719 (PI3Ki) or AZD8055 (mTORi) for 3h, and further subjected to phosphoproteomics analysis by mass spectrometry, following the protocol described in the S4 Appendix and previous work [16].

**Fig 3:**
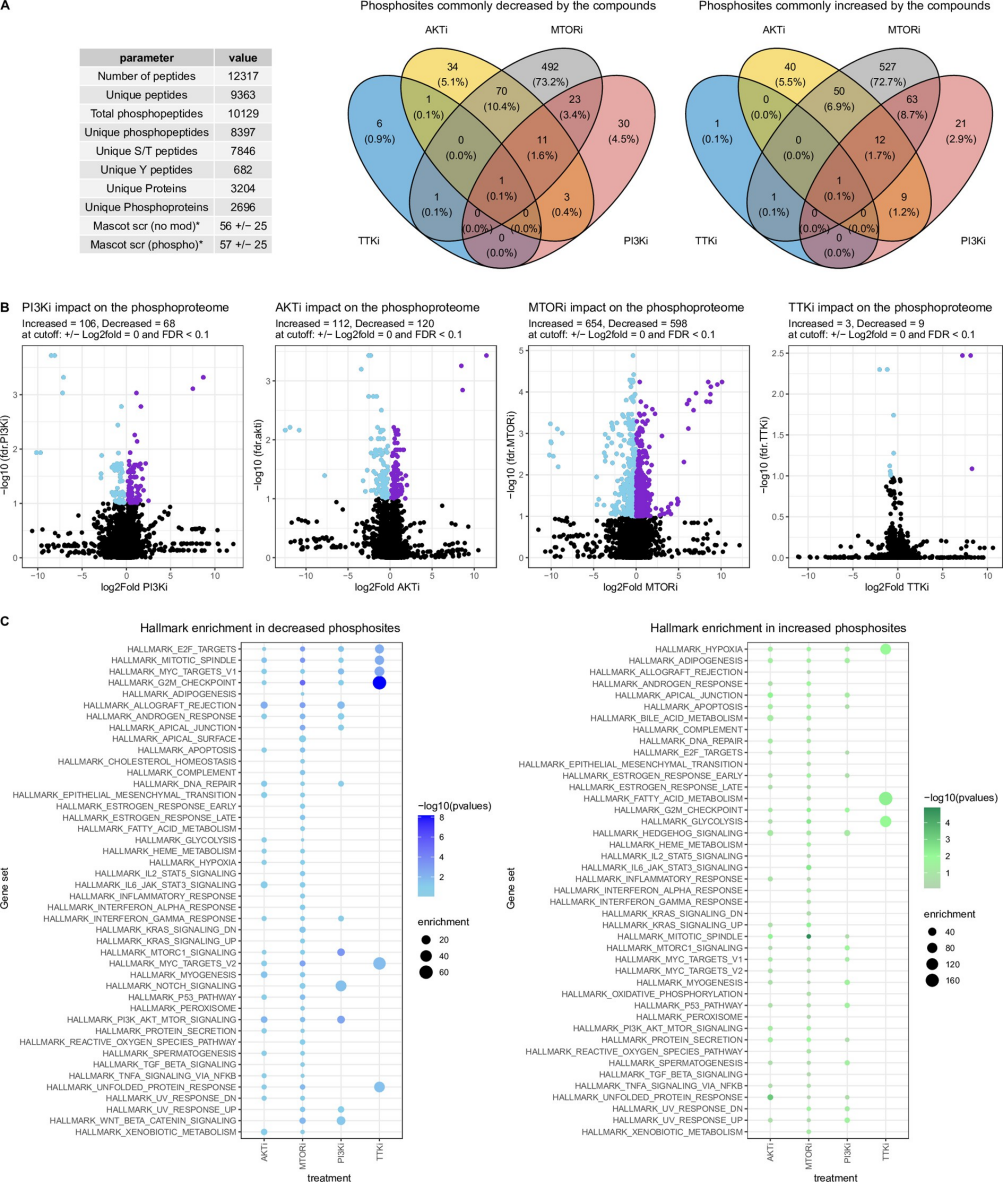
TTK and PI3K/AKT/mTOR pathway inhibitors impact common phosphorylation pathways. A. Summary of results of phosphoproteomics experiment. Venn diagrams show phosphopeptides commonly decreased or increased by the kinase inhibitors. B. Impact on the phosphoproteome as a function of the kinase inhibitors. Phosphopeptides increased or decreased significantly are highlighted in purple or sky blue, respectively. FDR values are p-values adjusted for multiple testing using the Benjamini-Hochberg method. C. Enrichment of hallmark gene sets from the Molecular Signature Database (MSigDB) considering phosphopeptides decreased (left panel) or increased (right panel) as a function of the compound treatments.

This analysis led to the identification and quantification of more than 8,000 phosphopeptides in biological and technical replicates (Fig 3A). As volcano plots in Fig 3B show, at the same threshold of statistical significance (adjusted *p*-value *<* 0.1), the MTORi caused the highest impact on the phosphoproteome (654 increased and 598 decreased phosphopeptides), whereas the TTKi only increased 3 and decreased 9 phosphosites compared to the control condition (Fig 3B). Treatments with PI3Ki and AKTi impacted more than 150 phosphorylation sites each. The larger impact on protein phosphorylation of PI3K/AKT/mTOR compared to TTK inhibition, suggests that TTK is unlikely to be upstream of the PI3K pathway.

**Fig 4:**
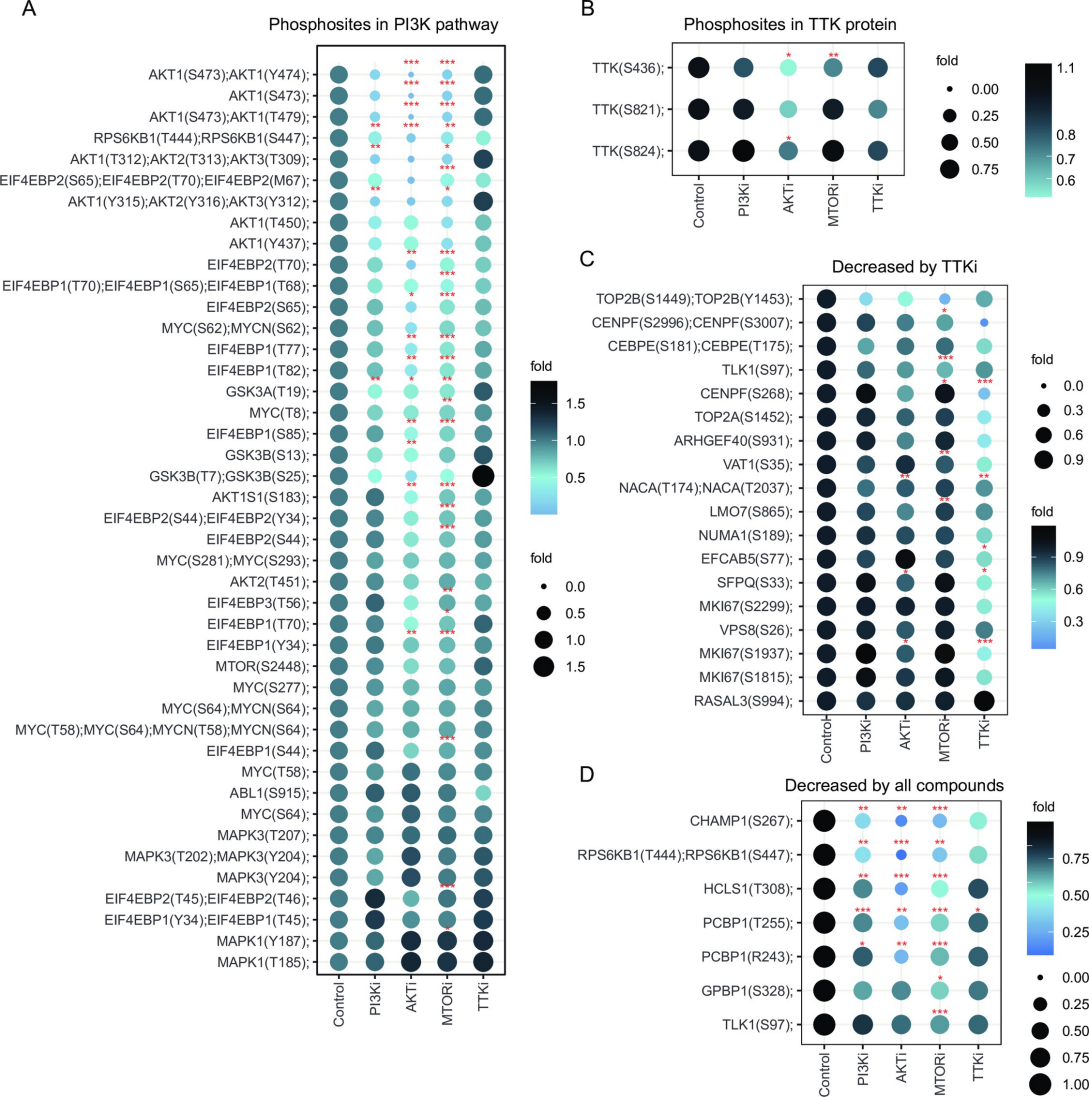
Phosphoproteomics of cells treated with kinase inhibitors supports a link between TTK and PI3K/AKT/mTOR signalling. Dot plots denote relative intensities of phosphosites as a function of treatment with the named kinase inhibitors, grouped by those linked to PI3K pathway activity (A), on TTK (B), decreased exclusively by the TTK inhibitor (C) or commonly decreased across all the treatments (D). FDR values are p-values adjusted for multiple testing using the Benjamini-Hochberg method.*∗ FDR <* 0.1, *∗ ∗ FDR <* 0.05, *∗ ∗ ∗ FDR <* 0.01, n = 4.

We found 7 and 4 phosphorylation sites commonly decreased or increased by all the kinase inhibitors, respectively (Fig 3A). Across the 7 phosphosites decreased, we highlight the double phosphorylated RPS6KB1 at T444 and S447, located in an autoinhibitory domain [33], the CDK1-mediated phosphorylation of CHAMP1 required for the attachment of spindle microtubules to the kinetochore [34] and the inhibition by phosphorylation of TLK1, which follows the generation of DNA double-stranded breaks during S phase as a DNA damage checkpoint (Fig 4D) [35]. These observations are consistent with the known roles of the PI3K pathway and the TTK mitotic kinase in the cell-cycle regulation and indicate a functional relationship between the two.

Furthermore, several markers of PI3K pathway activity, including multiple phosphorylation sites in AKT1/2, 4EPB1/2, PRAS40, GSK3A/B, MYC, were inhibited by PI3Ki, AKTi, MTORi but not by TTKi (Fig 4A), implying that TTK is not directly upstream of the PI3K pathway. Interestingly, we also found that the AKTi and mTORi significantly decreased the phosphorylation of TTK at S824 and S436 (Fig 4B), whose function are to regulate the activity of TTK in controlling cytoskeletal reorganisation [36]. Moreover, a pathway analysis using hallmark gene sets revealed an enrichment of the PI3K/AKT/mTOR signalling in phosphoproteins that decreased as a result of PI3Ki, AKTi or mTORi treatment, but not by TTKi (Fig 4C). In contrast, hallmark genes with roles in the G2M checkpoint and the mitotic spindle were enriched in phosphoproteins decreased by TTK as well as PI3K pathway inhibitors. Taken together, these observations are consistent with the finding obtained with the community detection algorithm and suggest that TTK phosphorylation and at least some TTK functions are downstream of the PI3K/AKT/mTOR network.

## Discussion

Studying the effects of kinase pathways dysregulation and over-activation on carcinogenesis has led to a better understanding of cancer biology and the development of therapies that target oncogenic kinase signalling. However, intrinsic and acquired resistance to treatments with kinase inhibitors and other drugs, which occur in most patients, is estimated to be responsible for 90% of cancer deaths [37]. Acquired resistance may occur through target modification, in which the drug target acquires further mutations that result in reduced drug binding, thus reducing the effectiveness of the treatment [38] or by the rewiring of kinase networks so that cancer cells use parallel or downstream pathways to proliferate [39]. The discovery of new members of oncogenic pathways, such as that of TTK and potentially PRKACA in the PI3K/AKT/mTOR pathway in this study, may help the identification of alternative targets for cancer treatment.

The two main aims of this study were first to assess whether it is possible to define sub-clusters of kinases that represent individual kinase signalling pathways by applying community detection algorithms to networks derived from empirical markers of kinase-kinase activity, and secondly, to assess whether such an approach can be used to identify new kinase members associated with canonical signalling pathways. Our study differs from most previous works on quantitative phosphoproteomics-based kinase network analysis, which focused on the prediction of kinase interactions using publicly available kinase-substrate databases, such as PhosphoSitePlus, Signor or Phospho.ELM (which draw information from systematic literature mining) [10,14]. A prior study [15] focused on identifying kinases that mediate crosstalk between different signalling pathways downstream of the activation of a receptor kinase. To do so, Narushima *et al* investigated a kinase-substrate network where interactions between distinct kinases are unweighted. Our study shows that, by harnessing newly available large scale phosphoproteomics datasets [16], community detection algorithms may also be used to identify new members of canonical signalling pathways.

Common limitations with many community detection algorithms include a “resolution limit” [40] and “near degeneracy” [41] [42] of the quality function. In the present analysis, communities were identified using modularity maximization at a single scale (or resolution) and a consensus partition was obtained by combining different locally optimal partitions using the approach in [25]. Appealing features of modularity maximization that may be useful in future investigations of mesoscale structure in kinase-substrate networks are that it has been generalized to weighted, directed, and time-dependent networks, and that it incorporates a user-specified “null model” that can be tailored to the application at hand.

One could extend this analysis by investigating communities at multiple scales, to identify smaller sets of densely connected kinases, and using other approaches to consensus clustering (e.g., that consider different null models) [43]. One could also consider different algorithms for optimising the quality function (e.g., Leiden [44]), or other community detection methods for a comparative analysis (e.g., random walk based approaches [45] [19]).

Overall, our study demonstrates that the study of community structure has the potential to reveal novel biological insights about cancer kinase signalling pathways. We found that (1) community assignments align well with established canonical pathways, (2) communities can reveal new components of known pathways, (3) wet lab experiments can validate the membership of the identified kinase to the known pathway, and provide evidence for whether it is activated downstream or upstream from it (i.e. TTK is likely downstream of PI3K/AKT/mTOR based on our results).

Future research in this field could include the application of network science techniques to investigate further questions in cell signalling, such as the relationship between kinase mesoscale network structure and an individual’s response to cancer treatment, where the latter might be measured by the proportion of cancer cells that cease to proliferate or decrease in viability post treatment. In the present paper, we investigated communities, which are the most commonly studied type of mesoscale structure. To try and gain further insights, one could also identify motifs in kinase networks [46] [47] and other types of mesoscale structure in networks that comprise different types of connections and/or exhibit temporal variation [48]. Ultimately, the hope is that such research can provide insights into the mechanisms of biochemical pathways and, as a result, improve our understanding of individual drug responses and advance methods for drug response prediction.

## Supporting information

S1 Appendix Partition and community robustness. (PDF)

S2 Appendix Analysis of kinase communities with strong links to MAP2K1 and MAPK1/3 and kinase communities with strong links to PIK3CA/AKT/mTOR signalling in ER+ breast cancer cells treated with rapamycin and oestrogen. (PDF)

S3 Appendix Analysis of networks constructed from edges with positive z-scores. (PDF)

S4 Appendix Phospho-proteomics data generation and analysis. (PDF)
