# Supplementary material for: Community detection in empirical kinase networks identifies new potential members of signalling pathways
Source: PLoS Comput Biol. 2023 Jun 23;19(6):e1010459. doi: 10.1371/journal.pcbi.1010459 (PMC10325051; doi:10.1371/journal.pcbi.1010459)
Supplement: S1 Appendix — (PDF) [file pcbi.1010459.s001.pdf]

S1 Appendix

Partition and community robustness

In this section we illustrate that the communities we analyse in the main text are robust to algorithmic stochasticity. We first compute ten different consensus partitions for each of the four networks using modularity maximization, the Louvain algorithm and the consensus approach in (2) (see Section 3.3 of main text for further detail). The mean and variance of the normalized mutual information (nMI) (1; 3) between partitions are as follows: $(\mu, \sigma) = (0.98, 0.023)$ for trametinib$^-$, $(\mu, \sigma) = (0.99, 0.01)$ for GDC0941$^-$, $(\mu, \sigma) = (0.97, 0.025)$ for AZD5363$^-$, and $(\mu, \sigma) = (0.96, 0.032)$ for GDC0994$^-$, where an nMI of 1 indicates that two partitions are identical. These results are obtained with 100 algorithmic runs within the consensus procedure. We recompute the consensus partition with a varying number of algorithmic runs (namely, 50, 100, and 200) and obtain the following nMI values: $(\mu, \sigma) = (0.98, 0.02)$ for trametinib$^-$, $(\mu, \sigma) = (1, 0)$ for GDC0941$^-$, $(\mu, \sigma) = (0.98, 0.011)$ for AZD5363$^-$, and $(\mu, \sigma) = (0.98, 0.012)$ for GDC0994$^-$.

It is important to note, however, that in our analysis we focus on communities that contain kinases of interest within these partitions, and not on all communities in the partition. The communities of interest are those that contain the main target of the inhibitor the cells were treated with, and are denoted trametinib$^-_{(\mathrm{MAP2K1})}$, GDC0941$^-_{(\mathrm{PIK3CA})}$, AZD5363$^-_{(\mathrm{AKT1/2})}$, GDC0994$^-_{(\mathrm{MAPK1/3})}$. We show the community content for each of these four communities in table 1. If we repeat the consensus procedure described in the main text ten times, the community content remains the same in almost all cases. The only exception is two nodes with smallest community strength in trametinib$^-_{(\mathrm{MAP2K1})}$ and one node with smallest community strength in GDC0994$^-_{(\mathrm{MAPK1/3})}$). Importantly, all other kinases (and in particular, the known canonical pathways shown in bold), remain as in table 1.

(a) trametinib$^{-}_{(MAP2K1)}$

Kinase	Community strength	Kinase	Community strength
AKT1.2	12.562		
PRKACA	8.318	**MAPK1.3**	2.75
TNK2	8.275	STK4	2.734
MTOR	7.820	PRKCI	2.704
**MAP2K1**	6.846	PIK3CB	2.577
PAK3	6.126	RPS6KB1	2.485
LATS1	6.000	PLK1	2.213
PIK3CA	5.817	CSNK1E	2.195
PAK1	4.040	PDGFRB	1.952
PRKACB	3.462	MAP3K1	1.836
TTK	3.321	YES1	1.786
**RPS6KA2**	3.057	PAK4	1.098
MINK1	2.825	STK3	1.092

(b) GDC0941$^{-}_{(PIK3CA)}$

Kinase	Community strength	Kinase	Community strength	Kinase	Community strength
**PIK3CA**	45.316	TNK2	14.143	PIK3CB	6.563
**AKT1.2**	42.634	MINK1	13.050	STK3	6.329
TTK	30.591	YES1	12.915	MAP4K5	6.080
**MTOR**	29.052	ABL1	12.393	PRKCI	6.027
PAK1	18.560	SRPK1	11.120	CSNK1E	4.997
LIMK1.2	18.088	PLK1	9.749	MAP2K1	4.685
PRKACA	15.715	PRKACB	8.495	**RPS6KB1**	4.22
LATS1	15.379	MAPKAPK2	7.817	RPS6KA2	4.085
PAK3	15.252	PAK4	7.367	PDGFRB	3.469
CIT	15.148	MAP3K1	6.700	MAPK14	3.115

(c) AZD5363$^{-}_{(AKT1/2)}$

Kinase	Community strength	Kinase	Community strength
**PIK3CA**	45.473		
**AKT1.2**	43.305	PLK1	7.485
LIMK1.2	30.722	PRKACB	7.081
TTK	28.068	MAPKAPK2	6.806
**MTOR**	27.884	CSNK1E	6.215
CIT	18.748	CDK2	5.146
PRKACA	14.637	PRKCI	5.012
LATS1	13.996	TNK2	4.925
PAK3	11.874	PAK1	4.875
MINK1	10.764	MAP4K5	4.475
ABL1	8.861	MAP3K1	4.222
PIK3CB	8.561	**RPS6KB1**	3.340
SRPK1	7.541	MAPK14	2.284

(d) GDC0994$^{-}_{(MAPK1/3)}$

Kinase	Community strength
**MAPK1.3**	4.240
TNK2	3.283
**MAP2K1**	2.047
PDGFRB	1.656
PRKCI	1.047
**ARAF**	0.732
CDK9	0.699
ROCK1.2	0.366

Table 1: Ground truth community content

References

1. Pedregosa F, Varoquaux G, Gramfort A, Michel V, Thirion B, Grisel O, Blondel M, Prettenhofer P, Weiss R, Dubourg V, Vanderplas J, Passos A, Cournapeau D, Brucher M, Perrot M, Duchesnay E. Scikit-learn: Machine Learning in Python Journal of Machine Learning Research. 2011;12:2825–2830

2. Lancichinetti A, Fortunato S. Consensus clustering in complex networks. Scientific Reports. 2012;2(1). doi:10.1038/srep00336.

3. Meilă M. Comparing clusterings—an information based distance J. Multivariate. Anal. 2007;98:873–895
