# Supplementary material for: Community detection in empirical kinase networks identifies new potential members of signalling pathways
Source: PLoS Comput Biol. 2023 Jun 23;19(6):e1010459. doi: 10.1371/journal.pcbi.1010459 (PMC10325051; doi:10.1371/journal.pcbi.1010459)
Supplement: S2 Appendix — (PDF) [file pcbi.1010459.s002.pdf]

S2 Appendix

Analysis of kinase communities with strong links to MAP2K1 and MAPK1/3

1 Community content

The application of community detection to the kinase interaction networks are as outlined in the main text. The results from the analysis of PI3K/AKT/MTOR networks are outlined in the main text. Here, we summarize the observations derived from the analysis of selected communities in trametinib^{-} and GDC0994^{-}, which we denote by trametinib$^{-}_{(\mathrm{MAP2K1})}$ and GDC0994$^{-}_{(\mathrm{MAPK1/3})}$ for the remainder of the article, contained both MAP2K1 (also known as MEK1) and MAPK1/3 (also known as ERK1/2). This is in line with the canonical understanding of RAF/MEK/ERK signaling pathway, which contains MAP2K1 (MEK1) and MAPK1/3 (ERK1/2), and which contributes to tumor progression by promoting cell proliferation and survival [1]. We however noted that ARAF, another constituent of this pathway was not part of trametinib$^{-}_{(\mathrm{MAP2K1})}$. The activity of the interactions between ARAF and the kinases assigned to both communities was increased in response to both inhibitors, except for that with MAPK1/3 and PDGFRB in GDC0994$^{-}_{(\mathrm{MAPK1/3})}$, with a z-score of -0.5 and -0.2 respectively (see heatmap in panel A of fig. 1), while

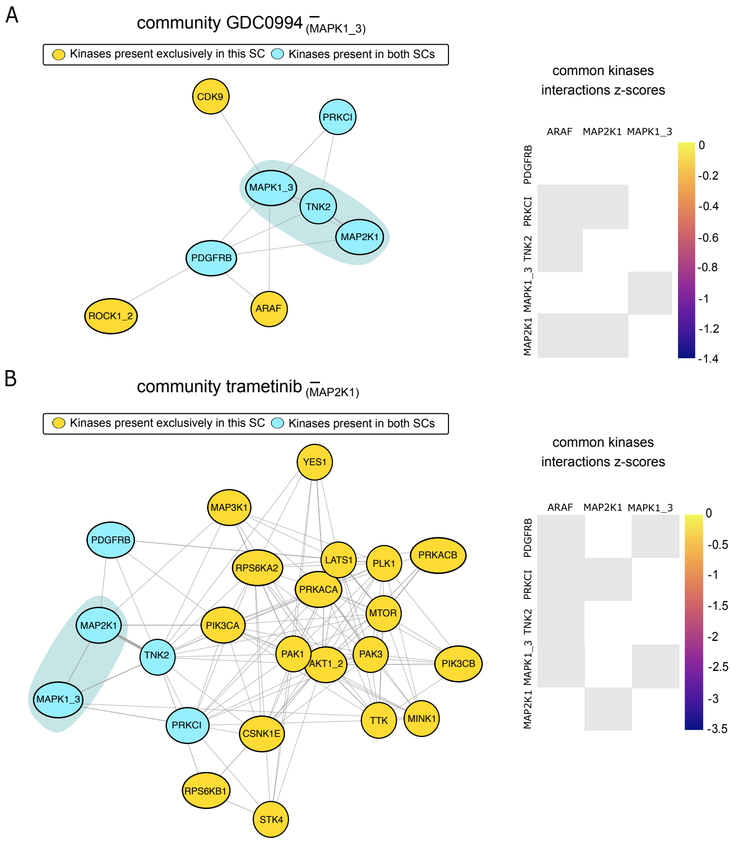

Fig 1. Kinase interactions of the selected communities in networks GDC0994^{-} and trametinib^{-}. Abbreviations: SC; selected community. On the left hand side of panel A and B the nodes and edges within the communities GDC0994$^{-}_{(\mathrm{MAPK1/3})}$ and trametinib$^{-}_{(\mathrm{MAP2K1})}$, respectively, are depicted, in which the blue nodes represent kinases present in both communities, and the yellow ones those exclusive to that community. The heatmaps on the right hand of each panel show the weights of the edges between well known members of the MEK/ERK pathway (x-axis) and the nodes present in both communities (y-axis), in each of the communities, in which a grey cell indicates that interaction is not present in that community.

the ARAF-MAP2K1 interaction in trametinib^{-} experienced a much smaller decrease in activity with a z-score of -0.17 (see heatmap in panel B of fig. 1).

Therefore, we conclude that ARAF is assigned to a different community than MAP2K1 and MAPK1/3 in the trametinib^{-} network because the activity of the ARAF interactions with the other kinases in trametinib$^{-}_{(\mathrm{MAP2K1})}$ is not significantly decreased in response to trametinib. Furthermore, only 5 kinases (highlighted in blue) were present across both trametinib$^{-}_{(\mathrm{MAP2K1})}$ and GDC0994$^{-}_{(\mathrm{MAPK1/3})}$(blue nodes in panels A and B of fig. 1), namely, MAP2K1, MAPK1/3, TNK2, PRKCI and PDGFRB. The presence of other 17 kinases (including PIK3CA, ATK, and MTOR) in trametinib$^{-}_{(\mathrm{MAP2K1})}$ (panel B in Fig S2) is consistent with the presence of MAP2K1 in GDC0941$^{-}_{(\mathrm{PIK3CA})}$(panel B in fig. 2), and highlight a potential cross-talk between the two pathways in the network as observed in other studies [2]. Thus, as with the communities returned by the analysis of cells treated with PI3K and AKT inhibitors, the networks selected communities in cells treated with MEK and ERK inhibitors contained canonical members of their respective pathways.

2 TNK2 may be at the crossroad of PI3K/AKT/mTOR signalling and MAP2K1

Further analysis of the data highlighted that the communities trametinib$^{-}_{(\mathrm{MAP2K1})}$, GDC0941$^{-}_{(\mathrm{PIK3CA})}$ and AZD5363$^{-}_{(\mathrm{AKT1/2})}$ are fairly similar in their content (Figure 2). All but two kinases in trametinib$^{-}_{(\mathrm{MAP2K1})}$ are present in GDC0941$^{-}_{(\mathrm{PIK3CA})}$, while four kinases are present in GDC0941$^{-}_{(\mathrm{PIK3CA})}$ and trametinib$^{-}_{(\mathrm{MAP2K1})}$, but not AZD5363$^{-}_{(\mathrm{AKT1/2})}$, that is MAP2K1, PDGFRB, YES1 and RPS6KA2. This analysis suggests that

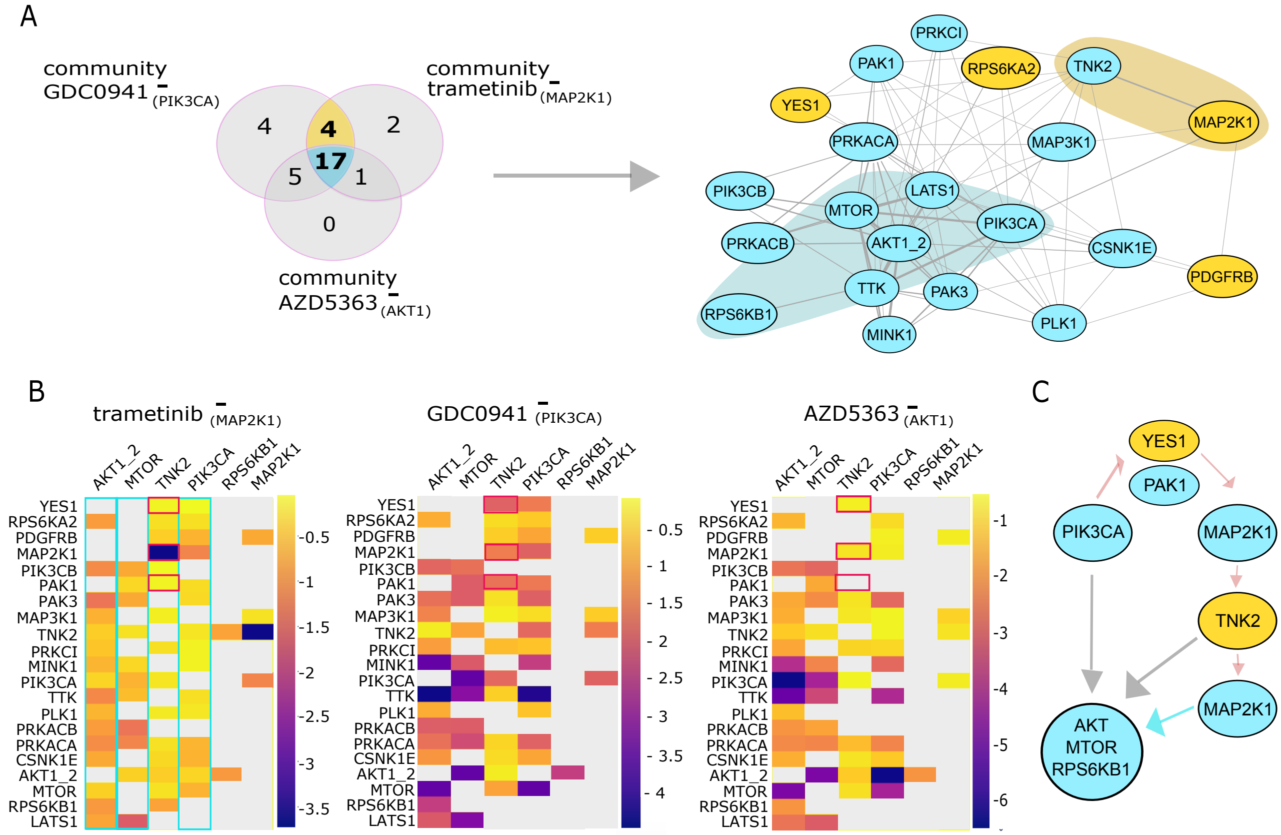

Fig 2. Intersection of the trametinib$^{-}_{(\mathrm{MAP2K1})}$, GDC0941$^{-}_{(\mathrm{PIK3CA})}$ and AZD5363$^{-}_{(\mathrm{AKT1/2})}$ communities. In the right hand side of panel A the kinases present across the three communities and their connections to each other are depicted in blue, while those present across trametinib$^{-}_{(\mathrm{MAP2K1})}$ and GDC0941$^{-}_{(\mathrm{PIK3CA})}$, but not AZD5363$^{-}_{(\mathrm{AKT1/2})}$ are shown in yellow. Panel B shows the z-scores of the kinase relationships shown in panel A, in each of the three communities. Panel C depicts a possible model of signalling between some of the kinases shown in the two other previous panels, based on the information on said panels (see text).

said kinases may be part of the MAP2K1 signaling pathway, which is influenced by PIK3CA, but not AKT1/2, activity. On the other hand, 19 kinases (shown in blue) are present across the three communities, including PIK3CA, AKT1/2, MTOR and RPS6KB1, none of which are present in GDC0994$^{-}_{(MAPK1/3)}$. Therefore, we estimate these are likely to be members of the PI3K/AKT/mTOR signaling pathway, which is influenced by MAP2K1, but not MAPK1/3, activity.

To get a better understanding of how PI3K/AKT signalling and MEK signalling may crosstalk, we took a closer look into the z-scores of the interactions of kinases attributed to MAP2K1 signalling and PI3K/AKT signalling in each of the networks of interest, as shown in panel B of Figure 4, from which we concluded that the interactions between the two pathways may be as depicted in panel C of Figure 4. First, it was observed that in response to MAP2K1 inhibition, the interactions of AKT1/2, mTOR and RPS6KB1 experienced a higher decrease in activity on average than those of PIK3CA, with average z-scores of -0.75, -0.71, -0.84 and -0.38, respectively, from which we conclude that it is more likely that MAP2K1 activates PI3K/AKT/mTOR signalling downstream of PIK3CA. Furthermore, the interaction between MAP2K1 and TNK2 displays the highest decrease in activity out of all kinase interactions in the trametinib^{-} network, with a z-score of -3.73, yet MAP2K1 inhibition does not significantly affect the activity of TNK2 interactions with other kinases in the community, as evidenced by z-scores closer to 0 in comparison, indicating that TNK2 is more likely to act upstream than downstream of MAP2K1. Additionally, the activity of the TNK2 interactions with YES1 and PAK1 is decreased in response to PIK3CA inhibition but not MAP2K1 inhibition (we consider a z-score close to 0 an indication that there is no change in activity) or AKT inhibition, from which it can be concluded that such interactions must be downstream PIK3CA but not MAP2K1, ergo it is likely that YES1 and PAK1 are activated downstream PIK3CA (note that PAK1 has been previously reported to be activated by PI3K/AKT signalling [3,4]) and TNK2 further downstream of those two kinases. If MAP2K1 is indeed downstream of TNK2, then MAP2K1 might activate AKT/mTOR signalling directly. However, if MAP2K1 acts upstream of TNK2, it is possible that it activates AKT/mTOR signalling through TNK2, as TNK2 has previously been stated to activate AKT [5,6].

3 The interactions between TTK and PI3K/AKT/mTOR signalling kinases are highly downregulated in response to mTOR inhibition in breast cancer cells

The results of our network computational analyses on in-house phosphoproteomics data from P31/FUJ cells treated with kinase inhibitors of the PI3K/AKT/mTOR pathway AZD5363 and GDC0941, as well as the additional wet-lab experiments performed, provide supporting evidence that TTK is activated downstream of PI3K/AKT/mTOR signalling in AML cells.

To validate our method and explore whether these results are applicable to other cancer cell lines, we reproduced these computational analyses on an external phosphoproteomics data from MCF-7 (breast cancer, positive for oestrogen receptor) cells treated with oestrogen and the mTOR inhibitor rapamycin [7]. The authors of this data provided the calculated difference in phosphorylation levels of 9500 phosphosites in MCF-7 cells treated with oestrogen and rapamycin compared to the untreated control MCF-7 cells in their supplementary information in each of the three replicates of their study. We averaged the values from the three replicates, and applied the KSEA method to this data to calculate the overall difference in phosphorylation levels of kinase interactions or edges (i.e. z-score) in the treated cells compared to the controls. We built a network from the edges with a negative z-score (i.e. inhibited in response to treatment), applied community detection to said network and selected the community containing the targeted kinase mTOR named rapamycin$^{-}_{(mTOR)}$.

We compared the absolute community strength centrality (i.e. the sum of the weight of the edges between a node and other nodes in its community) of the kinases in rapamycin$^{-}_{(mTOR)}$ and the kinases in the P31/FUJ cells communities GDC0941$^{-}_{(PIK3CA)}$ and AZD5363$^{-}_{(AKT1/2)}$, as shown on fig. 3 below. Interestingly, TTK has one of the highest absolute CSCs out of the kinases in rapamycin$^{-}_{(mTOR)}$ just like in GDC0941$^{-}_{(PIK3CA)}$ and AZD5363$^{-}_{(AKT1/2)}$, meaning that the interactions between TTK and PI3K/AKT/mTOR kinases are highly downregulated in response to mTOR inhibition. On the other hand, the absolute CSC of kinases PIK3CA, AKT1/2 and mTOR is not as high in rapamycin$^{-}_{(mTOR)}$ as it is in communities GDC0941$^{-}_{(PIK3CA)}$ and AZD5363$^{-}_{(AKT1/2)}$. This might be due to a counteractive effect of oestrogen on PI3K/AKT/mTOR inhibition by rapamycin, since binding of oestrogen to the ER generally increases the activity of PI3K/AKT/mTOR signalling [7].

These results show that TTK interacts with PI3K/AKT/mTOR signalling on ER+ breast cancer cells as well, and that our computational methods can be used on other phosphoproteomics datasets to better understand the effects of a treatment on a cell's signalling circuitry.

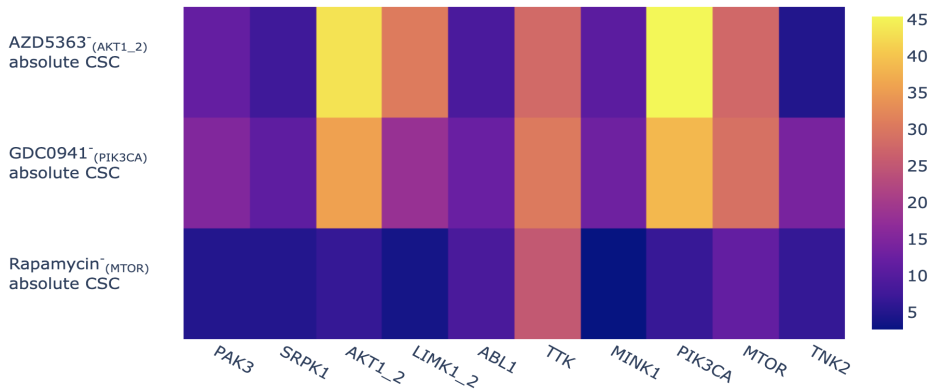

Fig 3. Comparisson of kinases community strength centrality across PI3K/AKT/mTOR communities in cells treated with PI3K/AKT/mTOR inhibitors. Abbreviations: CSC; community strength centrality. The heatmap shows the absolute community strength centrality of each of the kinases in the x-axis in each of the communities in the y-axis.

References

1. McCubrey JA, Steelman LS, Chappell WH, Abrams SL, Wong EWT, Chang F, et al. Roles of the Raf/MEK/ERK pathway in cell growth, malignant transformation and drug resistance. Biochimica et Biophysica Acta (BBA) - Molecular Cell Research. 2007;1773(8):1263–1284. doi:10.1016/j.bbamcr.2006.10.001.

2. Wang C, Cigliano A, Delogu S, Armbruster J, Dombrowski F, Evert M, Chen X, Calvisi D. Functional crosstalk between AKT/mTOR and Ras/MAPK pathways in hepatocarcinogenesis: Implications for the treatment of human liver cancer. Cell Cycle. 2013;12(13):1999–2010. doi:10.4161/cc.25099

3. Wu D-W, Wu T-C, Chen C-Y, Lee H. PAK1 Is a Novel Therapeutic Target in Tyrosine Kinase Inhibitor–Resistant Lung Adenocarcinoma Activated by the PI3K/AKT Signaling Regardless of EGFR Mutation. American Association for Cancer Research (AACR). 2016;22(21):5370–5382 doi:10.1158/1078-0432.ccr-15-2724

4. Yang Y, Du J, Hu Z, Liu J, Tian Y, Zhu Y, Wang L, Gu L. Activation of Rac1-PI3K/Akt is required for epidermal growth factor-induced PAK1 activation and cell migration in MDA-MB-231 breast cancer cells. Journal of Biomedical Research. 2011;25(4):237–245 doi:10.1016/s1674-8301(11)60032-8

5. Zhang A, Zhang R, Yang Z, Tian R. TNK2 promoted esophageal cancer progression via activating egfr-akt signaling Journal of Clinical Laboratory Analysis. 2021;35(5) doi:10.1002/jcla.23700

6. Mahajan K, Mahajan NP PI3K-independent AKT activation in cancers: A treasure trove for novel therapeutics Journal of Cellular Physiology. 2012;227(9):3178–3184 doi:10.1002/jcp.24065

7. Cuesta R, Gritsenko MA, Petyuk VA, Shukla AK, Tsai C, Liu T, McDermott JE, Holz MK. Phosphoproteome Analysis Reveals Estrogen-ER Pathway as a Modulator of mTOR Activity Via DEPTOR Journal of Cellular Physiology. 2012;227(9):3178–3184 doi:10.1002/jcp.24065
