# Supplementary material for: Community detection in empirical kinase networks identifies new potential members of signalling pathways
Source: PLoS Comput Biol. 2023 Jun 23;19(6):e1010459. doi: 10.1371/journal.pcbi.1010459 (PMC10325051; doi:10.1371/journal.pcbi.1010459)
Supplement: S3 Appendix — (PDF) [file pcbi.1010459.s003.pdf]

S3 Appendix

Analysis of networks constructed from edges with positive z-scores.

1 MEK/ERK inhibition upregulates p38 and JNK signalling possibly through the upregulation of ARAF and TNF signalling

We constructed two networks based on the edge enrichment of kinase-kinase relationships, measured as z-scores, of AML cells treated separately with the kinase inhibitors trametinib (a MAP2K1 inhibitor) and GDC0994 (a MAPK1/3 inhbitor). In this case, we only considered the edges with positive z-scores (i.e., kinase-kinase relationships that were up-regulated by the compounds), resulting in the networks trametinib$^+$ and GDC0994$^+$.

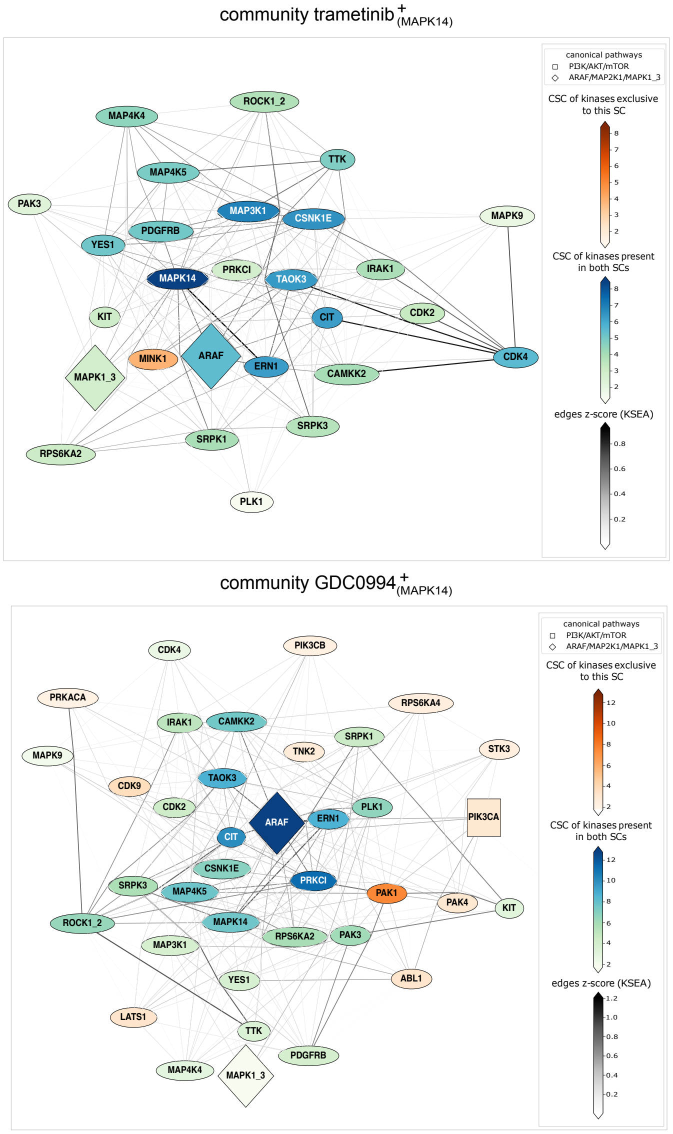

Fig 1. Comparison of kinases community strength centrality across MAPK14 communities in cells treated with MEK/ERK inhibitors. Abbreviations: SC; selected community, CSC; community strength centrality.

We applied community detection to both networks and selected the community with the highest average community strength centrality (CSC) in each, which we refer to as trametinib$^+_{(\mathrm{MAPK14})}$ and

$GDC0994^{+}_{(MAPK14)}$. We plotted these communities along with the CSC of each kinase and the z-scores of the edges in fig. 1 above. In both communities, ARAF has interactions with most, if not all, of the other kinases in the community, which are up-regulated to varying degrees. ARAF is inhibited by MAPK1/3 in a negative feedback loop, and MAPK1/3 is activated by MAP2K1 [1], therefore we conclude that this feedback loop is counteracted by MEK/ERK inhibition, resulting in an increase in ARAF activity. MAPK14(p38) is also highly up-regulated. Since there is a previous report of RAF-dependent regulation of MEKK/p38 [2], it is possible that ARAF increases MAP3K1 and MAPK14 activity in both communities.

MAPK9, a constituent of JNK signalling, as well as ERN1 and TAOK3, which interact with each other in the unfolded protein response (UPR) response in the endoplasmic reticulum, are also highly up-regulated. ERN1 might also indirectly up-regulate MAP3K1(also up-regulated), p38 and JNK signalling, as it leads to TNF production through the activation of NF-B signalling [4] [3], and TNF signalling activates both MAP3K1, p38 and MAPK9 (part of JNK signalling), while there is also evidence that p38 MAPK and JNK kinases promote TNF expression, therefore there might be a positive feedback loop between p38, MAPK9 and TNF [5]. MAP3K1 increases cell proliferation by upregulating the transcription factor AP-1 [6], which might explain why cyclin-dependent kinases (CDKs) CDK4 and CDK6 are up-regulated.

2 PI3K/AKT inhibition upregulates Raf/MEK/ERK signalling

We constructed two networks based on the edge enrichment of kinase-kinase relationships, measured as z-scores, of AML cells treated separately with the kinase inhibitors GDC0941 (a PI3K inhibitor) and GDC0994 (an AKT inhbitor). In this case, we only considered the edges with positive z-scores (i.e., kinase-kinase relationships that were up-regulated by the compounds), resulting in the networks $GDC0941^{+}$ and $AZD5363^{+}$. We applied community detection to both networks and selected the community with the highest average community strength centrality (CSC) in each, which we refer to as $GDC0941^{+}_{(MAPK14)}$ and $AZD5363^{+}_{(MAPK14)}$.

We plotted these communities along with the CSC of each kinase and the z-scores of the edges in fig. 2 below. In both communities the Raf/MEK/ERK signalling axis is up-regulated, which is in agreement with previous reports of Raf/MEK/ERK signalling being up-regulated in response to AKT inhibition in leukemia due to the weakening of the inhibitory binding of AKT to Raf1 [7]. Just like in the communities $trametinib^{+}_{(MAPK14)}$ and $GDC0994^{+}_{(MAPK14)}$ (see fig. 1), we observe an increase in the activity of the unfolded protein response (kinases ERN1 and TAOK3) and MAPK signalling (kinases MAPK14, MAP3K1, MAP4K4 and MAP4K5. Since the interaction between ARAF and MAPK14 is particularly down-regulated in both $GDC0941^{+}_{(MAPK14)}$ and $AZD5363^{+}_{(MAPK14)}$, it is highly likely that MAPK signalling is up-regulated through ARAF following the same mechanism described in section 1.

Interestingly, the interactions between ROCK1/2 and Raf/MEK/ERK, as well as MAPK kinases, are only slightly down-regulated or not at all, which indicates that ROCK1/2 activation is most likely not downstream of any of these kinases, but rather that they converge in downstream signalling (e.g. both the interaction between IRAK1 and ARAF, and IRAK1 and ROCK1/2 are strongly down-regulated). Furthermore, the interactions between ROCK1/2 and kinases PRKACA, IRAK1, CIT and TAOK3 are much more strongly down-regulated in $GDC0941^{+}_{(MAPK14)}$ than in $AZD5363^{+}_{(MAPK14)}$, indicating that ROCK1/2 activation might occur downstream of PI3K but not AKT. There is a previous report that PI3K negatively controls RhoA [8], which when bound to GTP is an upstream effector of ROCK, therefore it is possible that PI3K inhibition increases ROCK activity by attenuating the negative control of PI3K over RhoA. The interaction between ROCK1/2 and IRAK1 being up-regulated could indicate an increase in the activity of the pro-inflammatory pathway NF-B/NLRP3, since both kinases have been reported to act upstream of this pathway [9] [10].

On the other hand, the interactions between SRPK1, PDGFRB, MEK/ERK and MAPK kinases are much more strongly down-regulated in $AZD5363^{+}_{(MAPK14)}$ than in $GDC0941^{+}_{(MAPK14)}$, which indicates that SRPK1 and PDGFRB might be inhibited in an AKT-dependent manner. SRPK1 and PDGFRB contribute to angiogenesis, which usually helps the progression of tumors, therefore these might be an indication that AZD5363 needs to be administered along an angiogenesis inhibitor. In fact, a previous study found that combined AKT and SRPK1 inhibition is synergestic in acute lymphoblastic leukemia (i.e. a very close phenotype to the AML P31/FUJ cells of this study), which suggests that there is probably a regulatory feedback loop between AKT and SRPK1 [11]. However, since AZD5363 is a low specificity inhibitor [12], this increase in the activity of SRPK1 and PDGFRB interactions could also be due to off-target effects.

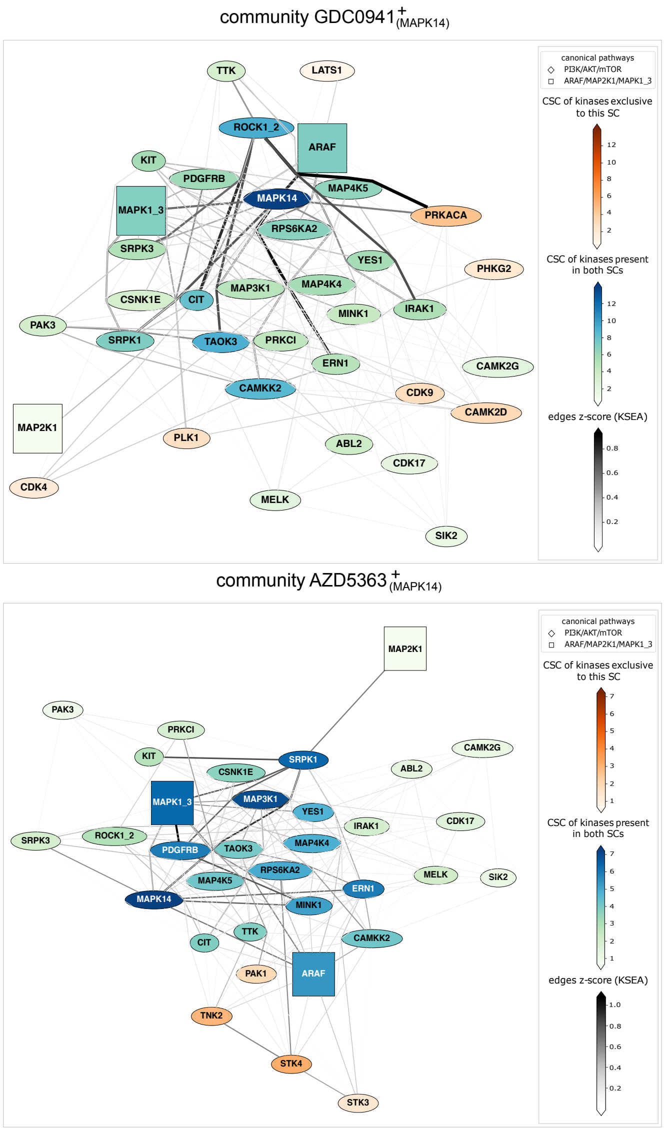

Fig 2. Comparison of kinases community strength centrality across MAPK14 communities in cells treated with PI3K/AKT/mTOR inhibitors.Abbreviations: SC; selected community, CSC; community strength centrality.

References

1. Barbosa R, Acevedo LA, Marmorstein R The MEK/ERK Network as a Therapeutic Target in Human Cancer. Mol Cancer Res 1 March 2021; 19 (3): 361–374. doi:https://doi.org/10.1158/1541-7786.MCR-20-0687

2. Pedrazza L, Schneider T, Bartrons R, et al. The ubiquitin ligase HERC1 regulates cell migration via RAF-dependent regulation of MKK3/p38 signaling. Sci Rep 2020; 10: 824. doi:https://doi.org/10.1038/s41598-020-57756-7

3. Read A, Schröder M The Unfolded Protein Response: An Overview. Biology. 2021; 10(5):384. doi:https://doi.org/10.3390/biology10050384

4. Hu P, Han Z, Couvillon AD, Kaufman RJ, John HE Autocrine Tumor Necrosis Factor Alpha Links Endoplasmic Reticulum Stress to the Membrane Death Receptor Pathway through IRE1-Mediated NF-B Activation and Down-Regulation of TRAF2 Expression Molecular and Cellular Biology. 2006; 26 (8): 3071-3084. doi:https://journals.asm.org/doi/abs/10.1128/MCB.26.8.3071-3084.2006

5. Sabio G, Davis RJ TNF and MAP kinase signalling pathways Seminars in Immunology. 2014; 26 (3): 237-245 doi:https://doi.org/10.1016/j.smim.2014.02.009

6. Zhou S, Niu R, Sun H, Kim S, Jin X, Yin J The MAP3K1/c-JUN signaling axis regulates glioblastoma stem cell invasion and tumor progression Biochemical and Biophysical Research Communications. 2022; 612: 188-195. doi:https://doi.org/10.1016/j.bbrc.2022.04.057

7. Wang J, Zhao Y, Kauss MA, Spindel S, Lian H Akt regulates vitamin D3-induced leukemia cell functional differentiation via Raf/MEK/ERK MAPK signaling. European Journal of Cell Biology 2009; 8 (2): 103-115. doi:https://doi.org/10.1016/j.ejcb.2008.05.003

8. Papakonstanti EA, Ridley AJ, Vanhaesebroeck B The p110 isoform of PI 3-kinase negatively controls RhoA and PTEN. The EMBO Journal 2007; 26: 3050-3061. doi:https://doi.org/10.1038/sj.emboj.7601763

9. Qian X, Yang L ROCK2 knockdown alleviates LPS-induced inflammatory injury and apoptosis of renal tubular epithelial cells via the NF-B/NLRP3 signaling pathway. Experimental and Therapeutic Medicine 2022; 24.3: 603. doi:https://doi.org/10.3892/etm.2022.11540

10. Dainichi T, Matsumoto R, Mostafa A, Kabashima K Immune Control by TRAF6-Mediated Pathways of Epithelial Cells in the EIME (Epithelial Immune Microenvironment). Front Immunol 2019; 10: 1107. doi:10.3389/fimmu.2019.01107

11. Siqueira RP et al Combined SRPK and AKT pharmacological inhibition is synergistic in T-cell acute lymphoblastic leukemia cells. Toxicol In Vitro 2020; 65:104777 doi:https://doi.org/10.1016/j.tiv.2020.104777.

12. Hijazi M, Smith R, Rajeeve V, et al. Reconstructing kinase network topologies from phosphoproteomics data reveals cancer-associated rewiring. Nat Biotechnol 2020; 38: 493–502. doi:https://doi.org/10.1038/s41587-019-0391-9
