# Supplementary material for: Community detection in empirical kinase networks identifies new potential members of signalling pathways
Source: PLoS Comput Biol. 2023 Jun 23;19(6):e1010459. doi: 10.1371/journal.pcbi.1010459 (PMC10325051; doi:10.1371/journal.pcbi.1010459)
Supplement: S4 Appendix — (PDF) [file pcbi.1010459.s004.pdf]

S4 Appendix
Phospho-proteomics data generation and analysis.

1 Sample preparation for phosphoproteomics analysis

10 mL of P31/FUJ cells suspension (1x106 cells/mL) were seeded in T25 flasks in RPMI medium supplemented with 10% FBS and 1% Pen/Strep and maintained in an incubator overnight at 37°C and 5% CO2. The following day, cells were treated with 1 µM CFI-402257 (TTKi), MK2206 (AKTi), BYL719 (PI3Ki) or AZD8055 (mTORi) for 3 hours.

Then cells were harvested by centrifugation at 500 x g at 4°C for 5 min, washed twice with ice-cold PBS supplemented with 1 mM Na3VO4 and 1 mM NaF, snap frozen and stored at -80°C until further processing. These experiments were performed in two biological replicates and two technical replicates. Cell pellets were lysed in urea buffer for 30 min. Cell lysates were homogenized by sonication and insoluble material was removed by centrifugation at 16,000 x g for 15 min at 4°C. Protein in the cell extracts was quantified by bicinchoninic acid (BCA) analysis. Subsequently, 250 μg of protein were reduced and alkylated by sequential incubation with 10 mM DTT and 17 mM iodoacetamide for 1 hour. The urea concentration was diluted to 2 M with 20 mM HEPES (pH 8.0) and 100 μL of conditioned trypsin beads (50% slurry of TLCK-trypsin) were added and the samples incubated for 16 hours at 37°C with agitation. Trypsin beads were removed by centrifugation at 2,000 x g for 5 min at 4°C.

Following trypsin digestion, peptide solutions were desalted using 10 mg OASIS-HLB cartridges. Briefly, OASIS cartridges were accommodated in a vacuum manifold (-5 mmHg), activated with 1 mL ACN and equilibrated with 1.5 mL washing solution (1% ACN, 0.1% TFA). After loading the samples, cartridges were washed twice with 0.75 mL of washing solution. Then peptides were eluted with 500 μL of glycolic acid buffer 1 (1 M glycolic acid, 50% ACN, 5% TFA) and subjected to phosphoenrichment. Phosphopeptides were enriched using TiO2 and sample volumes were normalized to 0.5 mL using glycolic acid buffer 2 (1 M glycolic acid, 80% ACN, 5% TFA), 25 μL of TiO2 beads (50% slurry in 1% TFA) were added to the peptide mixture, incubated for 5 min at room temperature with agitation and centrifuged for 30 s at 1,500 x g.

For each sample, 80% of the supernatant was transfer to fresh tubes and stored in ice and the remaining 20% used to re-suspend the bead pellets that were loaded into an empty prewashed PE-filtered spin-tips and packed by centrifugation at 1,500 x g for 3 min. After loading the remaining volume of the supernatant by centrifugation at 1,500 x g for 3 min, spin tips were sequentially washed with 100 μL of glycolic acid buffer 2, ammonium acetate buffer (100 mM ammonium acetate in 25% ACN) and 10% ACN by centrifugation for 3 min at 1,500 x g. For phosphopeptide recovery, the addition of 50 μL of 5% ammonium water followed by centrifugation for 3 min at 1,500 x g was repeated 4 times. Eluents were snap frozen in dry ice, dried in a speed vac and phosphopeptide pellets stored at -80°C.

2 Mass spectrometry

Phosphopeptide pellets were re-suspended in 18 μL of reconstitution buffer (20 fmol/μL enolase in 3% ACN, 0.1% TFA) and 5 μL were loaded onto an LC-MS/MS system consisting of a Dionex UltiMate 3000 RSLC directly coupled to an Orbitrap Q-Exactive Plus mass spectrometer. The LC system used mobile phases A (3% ACN; 0.1% FA) and B (100% ACN; 0.1% FA). Peptides were trapped in a µ-pre-column and separated in an analytical column (Acclaim PepMap 100). The following parameters were used: 3% to 23% B gradient for 60 min (phosphoproteomics) and a flow rate of 0.3 μL/min.

As they eluted from the nano-LC system, peptides were infused into the online connected Q-Exactive Plus system operating with a 2.1 s duty cycle. Acquisition of full scan survey spectra (m/z 375-1,500) with a 70,000 FWHM resolution was followed by data-dependent acquisition in which the 15 most intense ions were selected for HCD (higher energy collisional dissociation) and MS/MS scanning (200-2,000 m/z) with a resolution of 17,500 FWHM. A 30 s dynamic exclusion period was enabled with an exclusion list with 10 ppm mass window. Overall duty cycle generated chromatographic peaks of approximately 30 s at the base, which allowed the construction of extracted ion chromatograms (XICs) with at least 10 data points.

3 Peptide identification from tandem mass spectrometry data

Mascot Daemon 2.5.0 was used to automate peptide identification from MS data. Peak list files (MGFs) from RAW data were generated with Mascot Distiller v2.5.1.0 and loaded into the Mascot search engine (v2.5) in order to match MS/MS data to peptides [1]. The searches were performed against the SwissProt Database (SwissProt_Sep2014_2015_12.fasta) with an FDR of 1% and the following parameters: 2 trypsin missed cleavages, mass tolerance of ±10 ppm for the MS scans and ±25 mmu for the MS/MS scans, carbamidomethyl Cys as a fixed modification, pyroGlu on N-terminal Gln, oxidation of Met and phosphorylation on Ser, Thr and Tyr as variable modifications.

4 Peptide quantification from MS1 data

The in-house developed Pescal software was used for label-free peptide quantification [1], Phosphopeptides were quantified using a label-free method that uses XIC. Briefly, XICs for all identified peptides across all samples were constructed with ±7 ppm and ±2 min mass and retention time windows, respectively. Then, peptide intensity values were determined as the calculated peak areas of the constructed XICs. Peptide intensities for each sample were normalized to the sum of all peptide intensity values in the same sample.

References

1. Cutillas PR. Targeted in-Depth Quantification of Signaling Using Label-Free Mass Spectrometry. Methods in Enzymology 2017; 585: 245–68. doi: https://doi.org/10.1016/bs.mie.2016.09.021.
